# A ribosome profiling study of mRNA cleavage by the endonuclease RelE

**DOI:** 10.1093/nar/gkw944

**Published:** 2016-10-18

**Authors:** Jae-Yeon Hwang, Allen R. Buskirk

**Affiliations:** Department of Molecular Biology and Genetics, Johns Hopkins University School of Medicine, Baltimore, MD 21205, USA

## Abstract

Implicated in persistence and stress response pathways in bacteria, RelE shuts down protein synthesis by cleaving mRNA within the ribosomal A site. Structural and biochemical studies have shown that RelE cuts with some sequence specificity, which we further characterize here, and that it shows no activity outside the context of the ribosome. We obtained a global view of the effect of RelE on translation by ribosome profiling, observing that ribosomes accumulate on the 5′-end of genes through dynamic cycles of mRNA cleavage, ribosome rescue and initiation. Moreover, the addition of purified RelE to cell lysates shows promise as a method for generating ribosome footprints. In bacteria, profiling studies have suffered from relatively low resolution and have yielded no information on reading frame due to problems inherent to MNase digestion, the method used to degrade unprotected regions of mRNA. In contrast, we find that RelE yields precise 3′-ends that for the first time reveal reading frame in bacteria. Given that RelE has been shown to function in all three domains of life, RelE has potential to improve reading frame and shed light on A-site occupancy in ribosome profiling experiments more broadly.

## INTRODUCTION

Bacteria face enormous selective pressure from their physical and chemical environment and from competing micro-organisms. In response to this pressure, bacteria have evolved mechanisms to rapidly regulate gene expression, responding, for example, to a reduction in the levels of available nutrients by shutting down synthesis of ribosomes (the stringent response) (1,2). Another strategy bacteria use to deal with stress is to maintain a small fraction of the population in a dormant state that survives environmental insults and resumes growth when conditions improve (3). This latter strategy plays an important role in antibiotic resistance because dormant cells (known as persisters) are not killed, even at high antibiotic concentrations. Shutting down cellular protein synthesis is an essential step for both of these strategies.

RelE plays a direct and critical role in stress-response pathways and persistence by blocking translation. RelE is a member of the type II toxin-antitoxin family implicated in persister-cell formation (4). Overexpression of the RelE toxin causes a reversible inhibition of cell growth resembling the dormant state characteristic of persister cells (5). Growth resumes when RelE is neutralized by overexpression of its binding partner, the RelB anti-toxin. Under rich conditions, RelB is expressed at a slightly higher level, masking RelE activity, but under stress conditions, Lon protease degrades the more labile RelB anti-toxin, inducing RelE activity (6). Indeed, RelB was originally discovered in genetic screens involving nutrient starvation and named for its effects during the stringent response in which transcription of ribosomal RNA is inhibited through the accumulation of the alarmone ppGpp (7,8). Even under rich conditions, stochastic activation of RelE and related toxins is thought to be responsible for inducing a persister-like state in a small fraction of cells in culture (9).

Like at least 10 members of the type II toxin-antitoxin family in *E. coli*, RelE exerts its effects by cleaving mRNA and inducing translational arrest. RelE binds in the ribosomal A site and cleaves the RNA after the second nucleotide in the A-site codon (10). It is an unusual endonuclease in the sense that it does not cleave RNA outside of this context; the ribosome provides an environment essential to its catalytic activity. A catalytic mechanism has been proposed based on the x-ray crystal structure of RelE in a 70S ribosome complex and refined by follow-up kinetic studies (11–13). Initially, RelE was reported to be highly specific for select codons (CAG, UCG and the stop codon UAG) based on limited *in vitro* kinetic experiments (10). More recent studies by Woychik *et al.* suggest that the endonuclease has quite broad specificity *in vivo* with only a modest sequence preference, if any (14). Analyses of cleavage sites on a handful of highly-expressed genes identified many cleavage sites with only a modest preference for cleavage before G residues. These authors further made the puzzling observation that RelE cleaves primarily at the 5′-end of mRNAs, within about the first 100 codons; the mechanism underlying this polarity was not understood (14).

Here, we report a genome-wide characterization of protein synthesis in *E. coli* upon RelE overexpression. Distinct from the method of Woychik *et al.* who adapted RNA-seq to detect sites cleaved by another mRNA interferase, the MazF toxin (15), the ribosome profiling method that we employ directly reports on the position of ribosomes on mRNAs. This approach thus allows us to observe the effects of RelE on translation. We find that ribosome density is strongly enriched at the 5′-end of genes and we propose a model involving cycles of mRNA cleavage, rescue of stalled ribosomes and initiation that rationalizes the earlier observation of preferential RelE cleavage in the first 100 codons.

Further, we find that RelE can be used to improve the resolution and power of ribosome profiling in bacteria. As originally developed in yeast, ribosome profiling calls for digestion of naked mRNA with RNase I to generate ribosome footprints (16). As RNase I activity is inhibited by *E. coli* ribosomes (17), bacterial ribosome profiling studies have used MNase instead (18). Unfortunately, MNase is quite sequence specific (19), creating strong sequence bias at both the 5′- and 3′-ends of the ribosome footprint mRNA fragments, thus greatly biasing the pool of RNA fragments depending on their nucleotide sequence. Additionally, MNase generates a broad distribution of lengths of ribosome footprints (20), unlike the relatively homogeneous 28 nt footprint observed in ribosome profiling studies in yeast (16). This means that the position of the ribosome on the mRNA cannot be determined with sufficient precision to observe reading frame in ribosome profiling experiments in bacteria. Here, we show that the addition of purified RelE to *E. coli* cell lysates generates ribosome footprints that, for the first time, provide excellent information on the position and reading frame of the ribosome.

## MATERIALS AND METHODS

### Preparation of ribosome profiling and RNA-seq libraries

Libraries were prepared following our previously published protocol (21) with a few modifications. For the *in vivo* RelE expression experiments (the WT1 and RelE1 libraries), cells carrying pJC203 were grown with 12 μg/ml tetracycline. The pJC203 plasmid contains a pCDF origin, *tetR* marker, *araC* and the *relE* gene driven from the pBAD promoter; details are available upon request. Saturated cultures of *E. coli* MG1655 were diluted 1:100 into 400 ml of fresh LB media and grown at 37°C to an OD_600_ of 0.20. Arabinose was added to a final concentration of 0.2% to induce RelE expression and the cultures were grown for an additional 60 min. The cultures were then harvested by filtration and flash freezing; the wild-type culture had reached an OD_600_ of 0.83; the RelE-expressing cells only 0.41. mRNA fragments 20–40 nt in length were cloned for Ribo-seq and 40–60 nt fragments from total RNA cleaved by alkaline hydrolysis were cloned for RNA-seq.

For the *in vitro* RelE digestion experiments, wild-type cells were grown in MOPS media supplemented with 0.2% glucose, all 20 amino acids and other nutrients (Teknova). As above, an overnight culture was diluted and arabinose was added to a final concentration of 0.2% when the culture had reached an OD_600_ of 0.27. The cultures were grown for an additional 60 min and cells were harvested at a final OD_600_ of 1.4. (Strong Ser pauses were evident in these libraries, perhaps due to this late stage of growth). mRNA fragments 10–40 nt in length were cloned for Ribo-seq and RNA-seq (the WT2 and WT3 libraries). The RelE2 library was made from the same biological sample as the WT2 library; the only difference was the addition of 1 nmol of purified RelE to the MNase digest. RelE was purified following the procedure of Strobel *et al.* (12,13).

### Analyses of Ribo-seq and RNA-seq data

Following trimming of the linker sequence, reads that failed to align to rRNA and tRNA were aligned to the *E. coli* MG1655 genome NC_000913.3 using Bowtie 0.12.7 (22). Reads were required to map uniquely to the genome but were allowed two mismatches. Ribosome density maps were generated by assigning ribosome occupancy to the 3′-end of reads (21). Except where explicitly stated, genes with less than 0.1 reads per codon were excluded. Plots of average ribosome occupancy were computed by aligning genes at their start or stop codons and taking equally weighted averages of the ribosome density.

To compare ribosome density on different genes within a polycistronic message, we first needed to create a list of operons that are expressed from a single, non-overlapping message. The transcriptome of *E. coli* is remarkably complex (23): many operons overlap and multiple promoters often regulate subsets of genes within an operon. To define single transcription units, we started with roughly 4000 annotated operons in the RegulonDB database (24). We excluded all monocistronic genes as well as genes in polycistronic mRNAs in which the RNA-seq density varied by more than 5-fold (using RNA-seq data from the WT1 sample), since these genes probably contain multiple transcription units. We compared the ribosome density on the resulting 1131 genes in polycistronic mRNAs with more than 1 rpkm in the Ribo-seq and RNA-seq data.

Information on ribosomal reading frame was ascertained by mapping the 3′-end of ribosome footprints to the first, second or third nt of codons. All coding sequences were included (without any threshold for coverage), though reads within the first 30 nt and final 30 nt of each ORF were excluded. To compensate for RelE cleavage specificity, in the analyses of *prfB*, reads with 3′-ends mapping to the third sub-codon position on NNC codons were shifted to the second sub-codon position.

## RESULTS AND DISCUSSION

### Ribosomes are enriched at the 5′-end of genes in cells overexpressing RelE

To study the effects of RelE on protein synthesis *in vivo*, we overexpressed RelE in wild-type *E. coli* MG1655 cells and analyzed ribosome occupancy genome-wide using ribosome profiling. RelE expression from an *araBAD* promoter was induced with arabinose for 1 h beginning in early log phase. This protocol resulted in very high levels of RelE expression (one-quarter of all ribosome footprints map to the RelE mRNA) and, accordingly, we observed very pronounced effects on protein synthesis. The state that we describe represents an equilibrium state in which RelE activity has exerted its strong effects and cell growth has slowed or halted. Thus, by exaggerating the effects of RelE, relative to a more physiological activation, we obtain a clear picture of the scope and specificity of this endonuclease and its global effects on gene expression.

**Figure 1.**
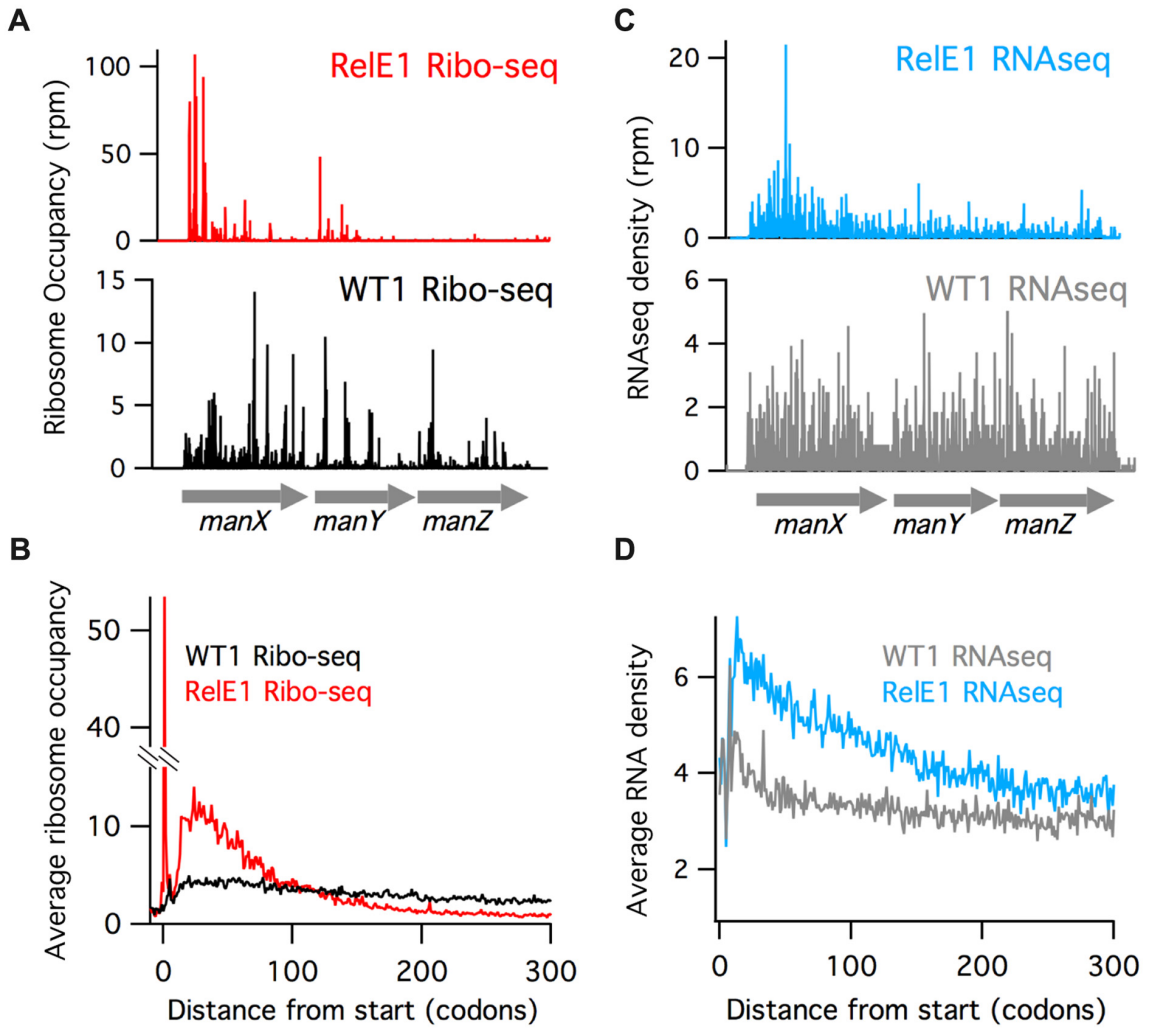
RelE overexpression causes ribosomes to accumulate at the 5′-end of genes. Gene models of (**A**) Ribo-seq and (**C**) RNA-seq data for the *manXYZ* operon in wild-type cells and a strain overexpressing RelE. Below, average (**B**) Ribo-seq and (**D**) RNA-seq density is shown for about 1000 genes aligned at their start codons.

The most striking effect of RelE overexpression is the dramatic enrichment of ribosome occupancy at the 5′-end of genes. In the model of the *manX* gene shown in Figure 1A, for example, far more ribosome footprints map to the 5′-end of the gene than the 3′-end (RelE1, red). In contrast, in the wild-type control (WT1, black), ribosome occupancy is distributed more or less equally across the *manX* gene. These observations hold true genome wide. In a plot of average ribosome occupancy of about 1000 genes, all over 1000 nt long, aligned at the start codon, ribosome coverage remains fairly constant in the wild-type control whereas in the RelE1 data ribosomes are highly enriched in the first 100 codons and depleted after the first 150 codons (Figure 1B). In addition, a very strong peak of ribosome density is observed at the start codon in the RelE1 data. This striking 5′-biased asymmetric distribution of ribosomes in the RelE overexpression strain suggests that the number of ribosomes completing synthesis of full-length proteins is strongly reduced. These observations are consistent with RelE's known ability to inhibit protein synthesis and arrest cell growth.

Given that RelE is an endonuclease that cleaves mRNA positioned inside the ribosomal A site, we also looked at steady-state levels of mRNA in RNA-seq libraries prepared from the WT and RelE overexpression samples. Here, again, we see enriched RNA density at the 5′-end of the *manX* gene (relative to the 3′-end) when RelE is overexpressed (Figure 1C, blue); in contrast, the coverage is relatively uniform in the wild-type control (grey). When the RNA-seq data from many genes is averaged together, a substantial increase in density at the 5′-end is observed in the RelE1 data (Figure 1D). These findings are nicely consistent with the ribosome profiling data.

To extend our analysis, we next evaluated gene expression across polycistronic transcripts and found that ribosome density decays not only across single genes but across operons as well. The *manXYZ* operon, for example, is expressed as a single transcription unit from a single promoter, a fact supported by equal levels of RNA density for the three genes in the WT1 RNA-seq data (Figure 1C, grey). Translation of all three genes is also observed in the WT1 Ribo-seq data (Figure 1A, black). In contrast, lower levels of RNA and far lower levels of ribosome footprints are observed for the downstream *manY* and*manZ* genes compared with *manX* upon RelE overexpression.

**Figure 2.**
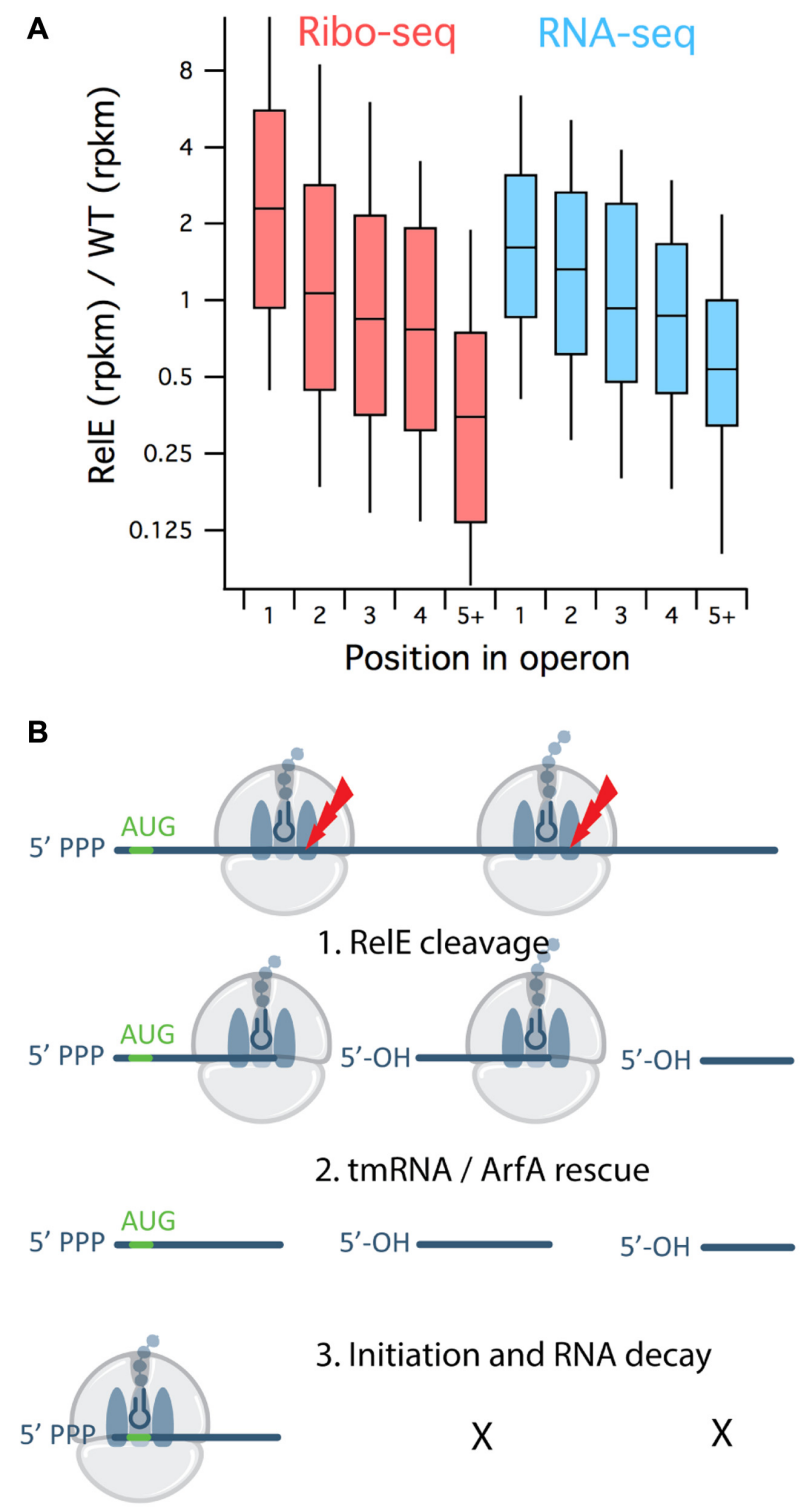
The dynamics of RelE cleavage, ribosome rescue and initiation explain the enriched ribosome density at the 5′-end of genes. (**A**) Upon RelE overexpression, Ribo-seq (red) and RNA-seq density (blue) is enriched in genes at the 5′-end of operons. The distribution of RelE1/WT1 ratios is shown for subsets of genes in polycistronic RNAs sorted by their position in operons. (**B**) Model for how RelE activity *in vivo* leads to accumulation of ribosomes at the 5′-end of genes.

To quantify these effects, we looked at the ribosome density for 1131 well-expressed genes found in 432 polycistronic operons. For each gene, we computed the ratio of ribosome density in the RelE1 sample over the density in the WT1 sample. The distribution of ratios are shown in Figure 2A, where genes are binned according to their position within the operon. Most genes that are at the first position in an operon have relatively more ribosome footprints in the RelE1 sample; e.g. the *manX* gene has 3-fold more (Figure 1A). In contrast, genes that lie downstream in the operon have relatively fewer ribosome footprints in the RelE1 sample: the *manZ* gene has 2-fold fewer. The difference between the distribution for genes in the first position versus genes in the fifth position (or further downstream) is statistically significant (Mann–Whitney *P*-value of 1.5 × 10^−20^). These broad differences are also observed at the RNA level (Mann–Whitney *P*-value of 1.3 × 10^−13^), though to a somewhat lesser extent. In summary, our data indicate that upon RelE overexpression, ribosomes are enriched not only at the 5′-end of the units of translation (open reading frames) but also at the 5′-end of units of transcription (polycistronic mRNAs).

We propose the following model for enrichment of ribosomes at the 5′-end of genes upon RelE expression (Figure 2B). As RelE begins to accumulate in the cell, it enters the A site of elongating ribosomes and cleaves the mRNA, thus preventing further cycles of elongation downstream. Stalled ribosomes are rescued by the tmRNA–SmpB complex or potentially by the backup ribosome rescue system comprised of ArfA and RF2, both of which lead to recycling of the ribosome subunits (25). Biochemical studies have shown that tmRNA reacts particularly well with ribosomes lacking mRNA downstream of the A site (26). In principle, the recycled subunits are then free to assemble on another transcript at the start codon to begin elongation again. If RelE cleaves the initiation complex with any efficiency, an enhanced start codon peak should be observed. If a few cycles of elongation take place before cleavage of the mRNA, ribosomes would be found enriched in the 5′ region of the coding sequence, decreasing in the 3′ direction. In this manner, cycles of mRNA cleavage, ribosome rescue and initiation could easily lead to an enrichment of ribosomes at the 5′-end of genes.

RNA decay mechanisms may also contribute to these observed polar effects. Following cleavage by RelE, the upstream RNA fragment will be protected from exonucleolytic degradation by the stalled ribosome at its 3′-end (27). In addition, the upstream fragment carries a 5′-triphosphate which is overall stabilizing (28). In contrast, the downstream fragment may be less occupied by ribosomes (as they will have run off) and may lack a start codon for another round of initiation. The 5′-hydroxyl group generated by RelE cleavage promotes recruitment of RNase E, facilitating further endonucleolytic cleavage and RNA decay.

Highlighting the importance of rescuing stalled ribosomes, we note that some of the rescue machinery is strongly upregulated in the RelE1 sample. We see less than 2-fold differences in tmRNA and SmpB levels between the RelE1 and WT1 libraries, consistent with the fact that these molecules are constitutively expressed in *Escherichia coli*. In contrast, steady-state levels for mRNA of the backup rescue factor ArfA are 15-fold higher in the RelE1 sample. The levels of synthesis of the ArfA protein (assessed by ribosome occupancy) are more than 80-fold increased. Interestingly, ArfA expression is regulated by tmRNA in an elegant feedback mechanism such that when the capacity of tmRNA is exceeded, ArfA accumulates (29,30). The fact that ArfA is strongly upregulated in cells treated with RelE suggests that the tmRNA system is overwhelmed and that cells are responding to the need for more ribosome rescue activity by upregulating ArfA expression.

As further support of our model, we note that in a previous study of RelE cleavage *in vivo*, cleavage sites were over-represented at the 5′-end of genes, although no explanation for this observation was provided (14). We argue that this observation is directly related to our observation of enrichment of ribosomes at the 5′-end of genes. The reloading of ribosomes at start codons, followed by a few rounds of elongation prior to additional cleavage events, ties these observations together: RelE is targeted to the 5′-end of genes because that is where ribosomes accumulate over time. Taken together, our findings support a model in which mRNA cleavage by RelE, ribosome recycling and initiation enrich ribosome occupancy at the 5′-end of genes.

### RelE predominantly cleaves mRNA after the second nucleotide in empty A sites

The ribosome profiling data obtained from cells overexpressing the RelE endonuclease provide information about cleavage sites genome-wide. In a sense, this information is indirect; we measure ribosome footprints and not the products of RelE cleavage themselves. In preparing the footprints, another nuclease (MNase) is used to digest upstream mRNA that is unprotected by ribosomes. The MNase treatment could interfere with our ability to detect RelE cleavage events if a significant fraction of mRNA fragments are generated by MNase alone.

**Figure 3.**
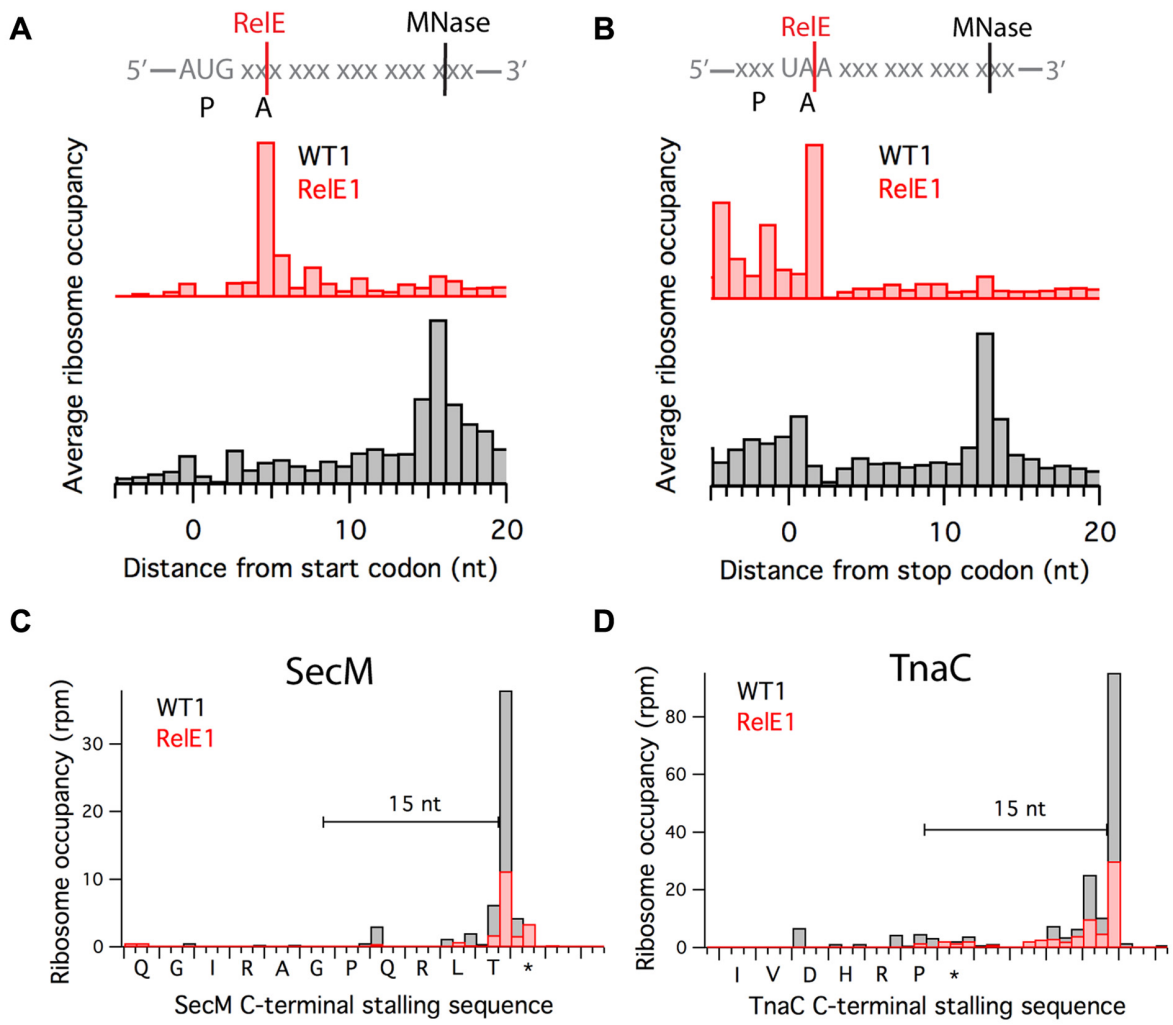
RelE cleaves mRNA in empty ribosomal A sites. Average Ribo-seq density at (**A**) start and (**B**) stop codons for Ribo-seq data from wild-type cells or cells overexpressing RelE; WT1 and RelE1 are shown at different scales. Gene models of two nascent peptide-mediated stalling sites: (**C**) SecM, where Pro-tRNA is bound in the A site and (**D**) TnaC, where the A site is filled by RF2.

We find that for the vast majority of the footprints, the 3′-end is generated by RelE cleavage and not MNase. This is likely due to the high concentration of RelE produced in the cell over the hour-long induction period. Support for the idea that most ribosome footprints are generated by RelE is seen from comparisons of the position of the start codon peak in plots of average ribosome occupancy (Figure 3A). In the WT1 control sample, MNase digests mRNA back to the 3′-boundary of the ribosome, 15 nt downstream of the first nucleotide in the start codon (black), corresponding to what we have observed in many bacterial profiling libraries generated with MNase (21). In contrast, the start codon peak in the RelE1 data is 4 nt downstream of the first nucleotide in the start codon (red).

The same phenomenon is observed at stop codon peaks in plots of average ribosome occupancy (Figure 3B). During termination, the stop codons UAG, UGA or UAA are positioned in the ribosomal A site. In the WT1 sample, MNase digests back to the 3′-boundary of the ribosome, 12 nt downstream of the first nucleotide in the stop codon (black). In contrast, RelE cleaves after the second nt in the stop codon (red) in the A site. Together, the data at start and stop peaks show that RelE cleaves predominantly after the second nucleotide in the A-site codon and that most ribosome footprints in these samples are generated by RelE cleavage. This pattern of cleavage is broadly consistent with previous enzymatic, structural and primer extension studies of RelE activity (10,11,14).

In examining peaks at other well-characterized ribosome pausing sites (specific sites rather than gene averages), we observed an interesting exception to the A-site cleavage patterns seen at start and stop codons. The SecM and TnaC polypeptides interact with the ribosome to inhibit their own synthesis in response to specific cellular signals (31). The ribosome is known to stall at the RAGP sequence in SecM, e.g. with the Gly codon in the P site and unreactive Pro-tRNA bound in the A site (32,33). A strong (black) peak 15 nt downstream of the Gly codon in the WT1 sample corresponds to this well-understood pausing event when the samples are treated only with MNase (Figure 3C). In the RelE1 data, the expected peak after the second nucleotide in the Pro codon is not observed; instead, the strongest peak overlaps with the one observed in the WT1 sample 15 nt downstream of the Gly codon. This exception makes an important point. These data suggest that RelE is unable to cleave the Pro codon in the A site in stalled SecM complexes, presumably because the Pro-tRNA is stably bound there and blocks RelE entry. Similarly, ribosome stalling at the C-terminus of the TnaC peptide (Figure 3D) is observed in the WT1 data with a strong peak 15 nt downstream of the final Pro codon. In the RelE1 data, a peak might be expected within the stop codon positioned in the A site, but none is observed. We argue that RelE is blocked by bound release factor 2 that is recruited but cannot hydrolyze the nascent peptide (34). These findings suggest that RelE is capable of competing with aminoacyl–tRNA and release factors for access to the A site during normal elongation and termination, but that stably-bound factors block RelE activity.

### *In vitro* digestion with RelE reveals the reading frame

It has been impossible to detect reading frame in ribosome profiling studies in bacteria. This limitation arises in large part from the nuclease, MNase, used to prepare samples. The principle problem is that the nuclease has substantial sequence specificities that can result in one or more nucleotides remaining undigested at the boundaries of the ribosome. Given the precision with which RelE cleaves after the second nucleotide in the A site when expressed *in vivo*, we hypothesized that RelE might prove to be a useful tool in ribosome profiling, generating more reliable 3′-ends and improving the resolution at which the position of the ribosome can be determined. With this goal in mind, we purified RelE and added it to cell lysates under our regular digestion conditions *in vitro*; MNase was also included at the regular concentration to digest mRNA on the 5′-boundary of the ribosome. Ribosome footprints 10–40 nt in length were then cloned and sequenced following our usual protocol. Because it cleaves in the A site, RelE produces mRNA fragments shorter than those produced by MNase cleavage alone (Supplementary Figure S1).

**Figure 4.**
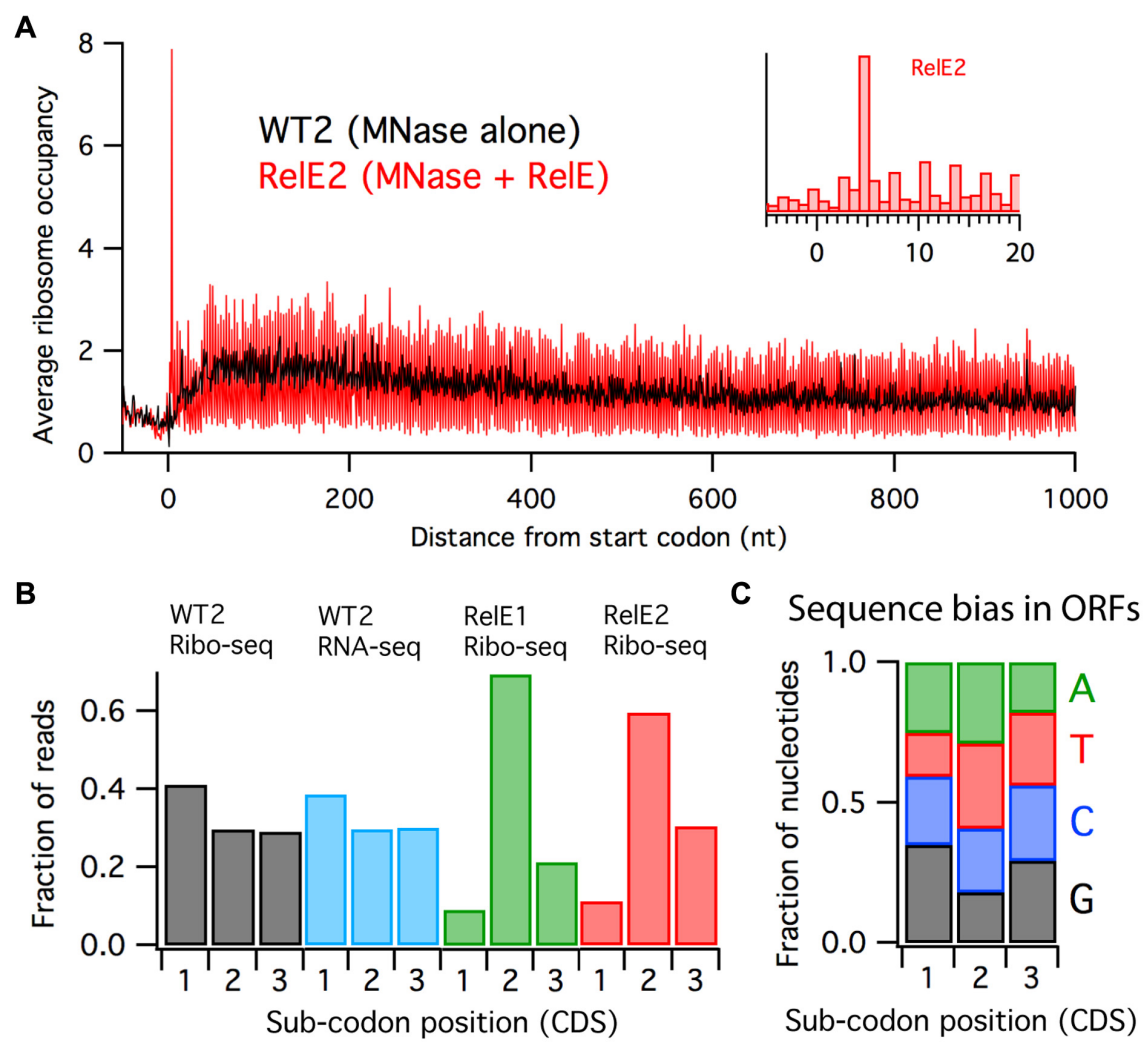
RelE-treated samples reveal ribosomal reading frame. (**A**) Average Ribo-seq density aligned at start codons for a wild-type sample where RNA was digested *in vitro* by MNase alone (WT2) or by MNase and purified RelE protein (RelE2). Inset: detail of RelE2 signal at the start codon. (**B**) Fraction of Ribo-seq and RNA-seq reads with 3′-ends that map to each position within codons. (**C**) Sequence bias in all ORFs in the MG1655 genome.

The outcome of RelE digestion *in vitro* can be seen in plots of average ribosome occupancy across many genes aligned at start codons (Figure 4A). A robust start codon peak is found at +4 and not +15 in the *in vitro*-digested RelE2 sample (inset), exactly as observed when RelE is overexpressed *in vivo* (RelE1, Figure 3A). On the other hand, the *in vitro* RelE-digested data (RelE2) lack the strong enrichment of ribosome density at the 5′-end of genes that we observed in the *in vivo* RelE-digested data (RelE1). This observation is nicely consistent with our model for the 5′ accumulation of reads in the RelE1 sample: on-going translation is necessary for ribosomes to accumulate on the 5′-ends of genes following RelE cleavage, and this phenomenon is not seen *in vitro* because elongation is arrested by chloramphenicol and initiation is inhibited by dilution of the necessary factors. These observations provide further evidence that the enrichment of ribosome occupancy at the 5′-end of genes *in vivo* is due to the dynamics of cleavage, rescue and initiation and not RelE activity per se.

A second striking observation in these data is the wide vertical spread in the RelE2 signal relative to that observed in the wild-type (WT2) library prepared from the same biological sample using only MNase (Figure 4A). This vertical spread is indicative of 3 nt periodicity, some portion of which reflects reading frame.

To begin to consider this question, we first calculated the average ribosome density at all three sub-codon positions (1,2 and 3) in open reading frames throughout the genome. The WT2 library shows slightly higher density at the first nucleotide (40%) than the other two nucleotides (30% each, Figure 4B, black). While it is tempting to attribute this 3 nt periodicity to reading frame, we have found that RNA-seq libraries prepared by digestion of total RNA with low concentrations of MNase are similar –ribosome occupancy is enriched at the first sub-codon position in ORFs (Figure 4B, blue). We argue that the sequence specificity of MNase coupled with the nucleotide bias in ORFs explains this observation. Indeed, it is known that MNase cleaves preferentially before A and T residues (19), and analysis of nucleotide bias in ORFs reveals that these two nucleotides are enriched at position two in codons (Figure 4C). Cleavage before position two makes the 3′-end of footprints align with the first sub-codon position. Given that ribosome occupancy is assigned using the 3′-end of footprints, ribosome density appears higher at the first position (40%) than the other two (30% each). This effect is lacking in footprints mapping to 3′-untranslated regions (where the reading frame of reference is defined by the preceding ORF), because there is no nucleotide bias in the 3′-UTR (Supplementary Figure S2). These data show that the small degree of 3 nt periodicity in profiling data produced with the regular protocol arises from MNase specificity and not the reading frame of the ribosome.

In contrast, libraries treated with RelE either *in vivo* or *in vitro* clearly show the strongest density at sub-codon position two (Figure 4B), corresponding to cleavage before the third nucleotide in the A-site codon. This specificity is similar to what we observed at both start and stop codons in average ribosome density plots (Figure 3). In the *in vitro*-digested sample (RelE2), roughly 60% of footprints map to the second sub-codon position; 30% map to the third while only about 10% map to the first position. As expected, reading frame is lacking in non-translated regions (Supplementary Figure S2). The large difference between the density at the first two positions explains the large vertical spread observed in Figure 4A. In the RelE overexpression library (RelE1), the difference is even more exaggerated: 70% of footprints map to the second position, while only 20% map to position three and 10% to position one. These libraries provide the first reliable information about ribosome reading frame in bacterial ribosome profiling data.

### RelE prefers to cleave after C and before G

**Figure 5.**
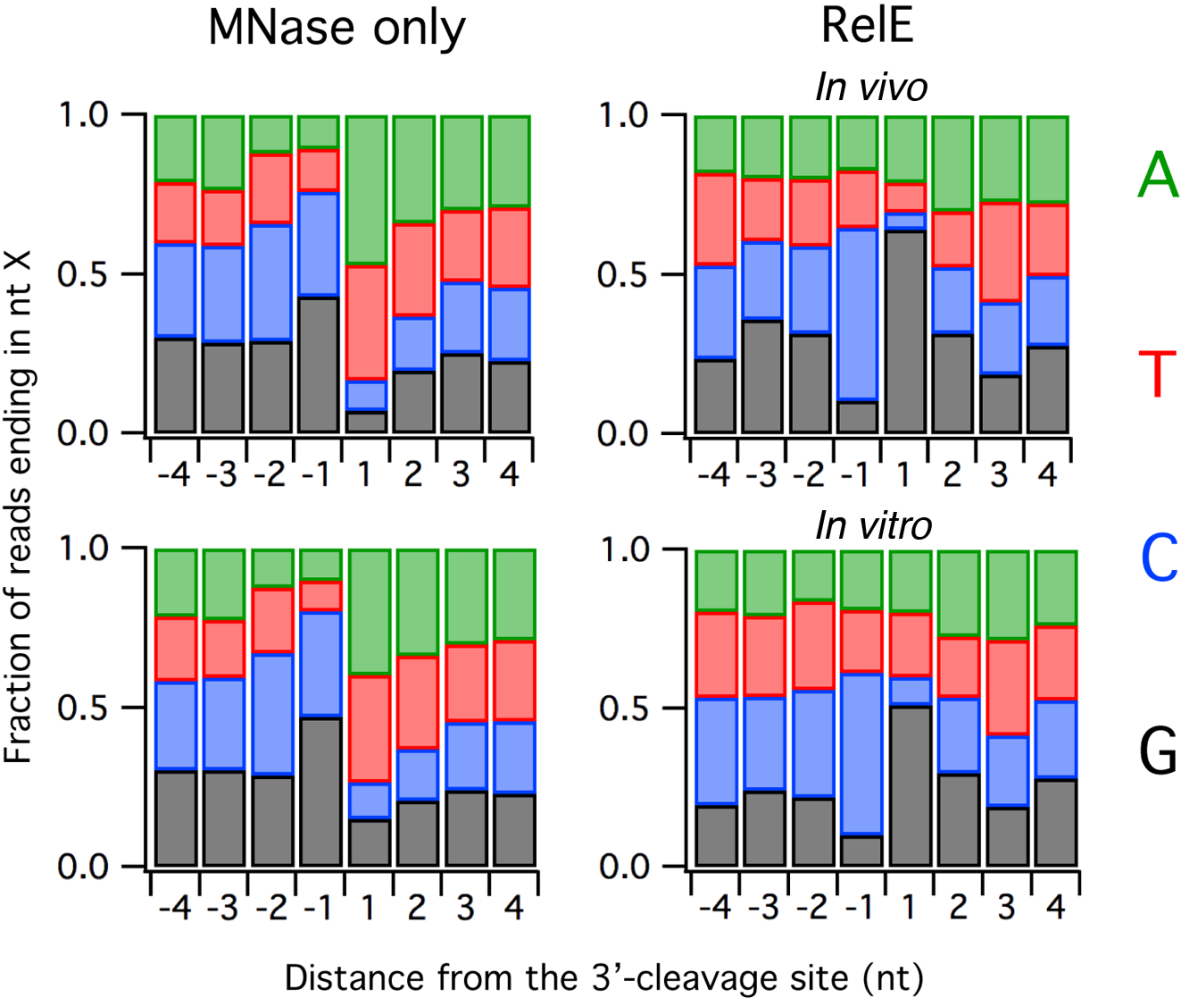
Sequence bias at the 3′-end of ribosome footprints due to nuclease specificity. Left: two samples digested with MNase *in vitro*. Right: the RelE1 library with RelE overexpressed *in vivo* (top) and the RelE2 library in which purified RelE was used to digest RNA *in vitro* (bottom). MNase was also used to prepare to these two RelE libraries.

By analyzing the nucleotide bias at the 3′-ends of ribosome footprints in the different experiments, we are able to obtain some insight into the specificities of the MNase and RelE enzymes. Nuclease specificity is not the only contributor to this bias; sequence bias in open reading frames, enrichment of ribosomes at specific sites on transcripts and cloning or sequencing artifacts may also play a role. But by comparing RelE-derived footprints with MNase-derived footprints and keeping the other factors constant, we were able to observe some interesting trends that can be attributed to nuclease specificity. As others have shown, MNase preferentially cleaves before the nucleotides A and T; in our data we observe that A and T are strongly enriched downstream of the cleavage site, both at +1 nt and to a lesser extent +2 nt (Figure 5, left). In contrast, A and T are underrepresented upstream of the cleavage site, both at −1 nt and to a lesser extent −2 nt. The sequence preferences of RelE are very different from those of MNase: C is preferred at the −1 position while G is selected against (Figure 5, right). Following the cleavage site, G is strongly preferred and C is selected against. These tendencies hold true whether RelE digestion occurs *in vivo* (RelE1) or *in vitro* (RelE2).

Although initial reports of RelE activity described the endonuclease as highly specific for a few codons, additional studies revealed a more relaxed specificity. The preference for G after the cleavage site was anticipated by the kinetic studies of Ehrenberg *et al.*, who found that k_cat_/K_M_ values for RelE cleavage were markedly higher for codons ending in G (10). Woychik *et al.* also reported a strong preference for cleavage before G in five highly expressed genes in *E. coli* (14). The x-ray crystal structure of RelE bound in the A site of 70S ribosomes offers a possible clue explaining this preference (11). The G in the third position of the A-site codon stacks on the base of C1054, a conserved 16S rRNA nucleotide in the decoding center. Direct contact of RelE residues with this G nucleotide are also possible.

In contrast to the good agreement of our data with these earlier studies regarding the specificity of the downstream nucleotide at the cleavage site, our observations of a marked preference upstream of the cleavage site are unexpected. There is little evidence in the literature of a strong bias for C (and against G) at the −1 position, raising the possibility that this effect is an artifact of ribosome profiling. We find, however, that RNA-seq libraries show no such bias when fragments were prepared by alkaline hydrolysis and then cloned and sequenced exactly as the profiling libraries (Supplementary Figure S3). This result suggests that the preference for C may be due to RelE cleavage itself rather than cloning and sequencing bias. The specificity of RelE cleavage has implications for its use in ribosome profiling: because it cleaves with some sequence specificity, some undesirable noise is introduced during the library preparation that may interfere with quantitative observation of ribosome pauses at the A site (Supplementary Figure S4). We argue, however, that this limitation does not affect the number of ribosomes observed per gene (Supplementary Figure S5), a common metric in profiling studies, and is offset by the advantages that RelE offers in terms of providing more precise information about the ribosome's position and reading frame. It is possible that related RelE enzymes from other organisms will either exhibit complementary or reduced specificities, thus increasing the generality of this approach.

### Refined RelE-derived ribosome density better reflects reading frame

**Figure 6.**
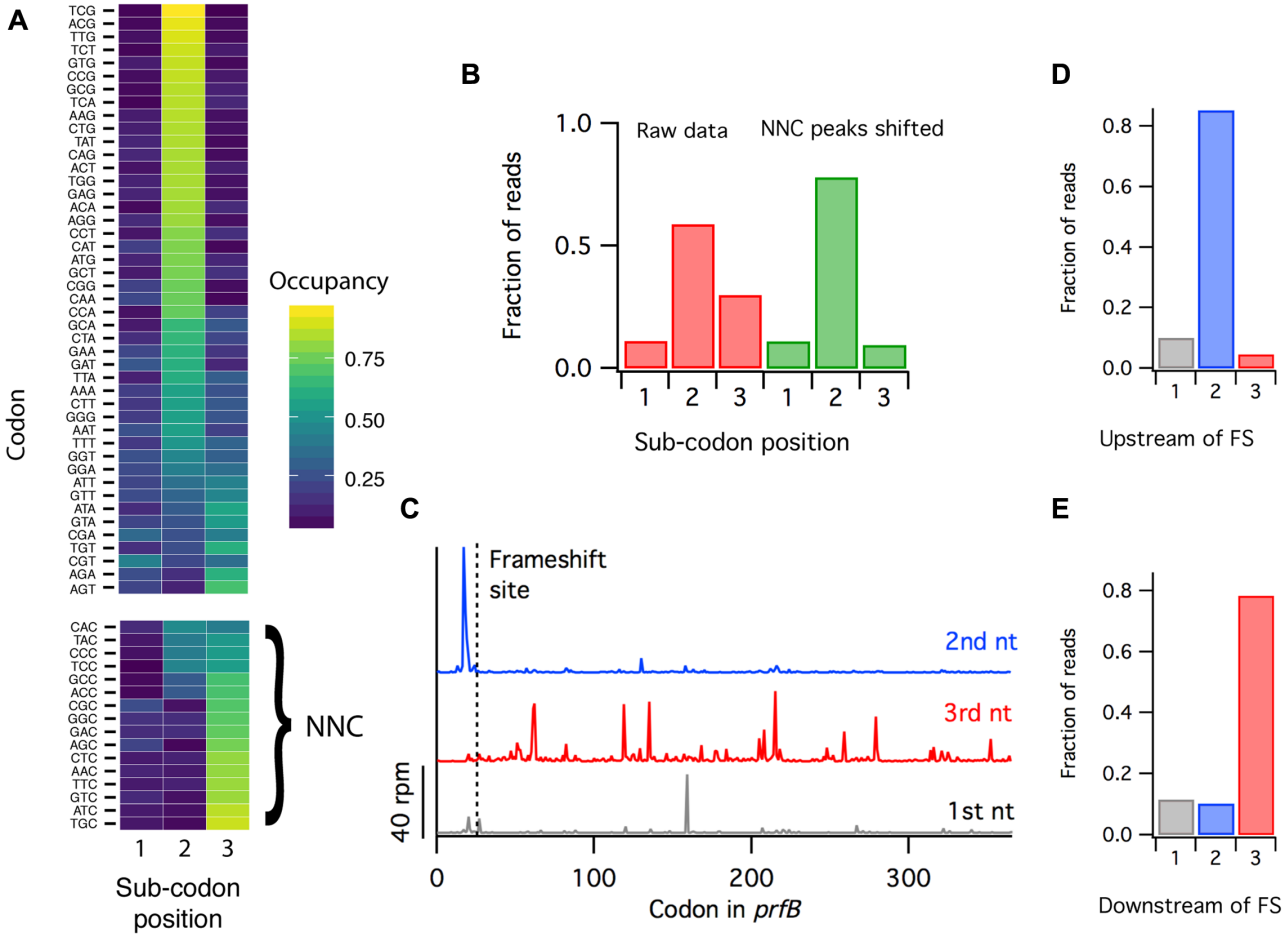
After nuclease bias is taken in to account, libraries generated by RelE cleavage reveal programmed frameshifting at the *prfB* gene. (**A**) Ribosome occupancy from the RelE2 (*in vitro*) sample at all 61 sense codons broken down by sub-codon position. (**B**) Fraction of reads mapping to each sub-codon position, before and after shifting the density at NNC codons back one nucleotide. (**C**) Model of the *prfB* gene containing a well-characterized +1 frameshift at the 28th codon, with the Ribo-seq signal split into three components, each representing data mapping to a single sub-codon position. (**D** and **E**) Fraction of reads mapping to each subcodon position before and after the +1 frameshifting site in *prfB*.

Because the sequence specificity of RelE alters the pattern of cleavage in predictable ways, we can refine the data computationally and obtain even better information regarding reading frame. Looking at average ribosome occupancy at sense codons positioned in the A site, we find that most codons are cleaved after the second nucleotide, as expected from the analyses of start and stop codons above. In the heat map shown in Figure 6A, most codons have the strongest signal at position two, corresponding to cleavage after the second nucleotide. A subset of codons has elevated density at position 3; all of the codons ending in C have more cleavage after the third sub-codon position than after the second position. Given the specificity depicted in Figure 5, where cleavage after C is preferred and cleavage before C is strongly avoided, it makes sense that NNC codons are cleaved after the third position, between the A site codon and the codon downstream. To compensate for this cleavage bias, we shifted the ribosome density at all NNC codons from the third nucleotide to the second. This improved the signal at the second position from about 60% to about 80%, with 10% remaining at position one and 10% at position three (Figure 6B). The fact that the signal at position three was reduced from 30% to 10% suggests that two-thirds of reads at this position arise from NNC codons. Given what we know about sequence specificity, a more sophisticated algorithm could be employed in the future to realign ribosome density to optimize information about reading frame.

This simple shifting of density on NNC codons from the third to second position results in substantial improvements in reading frame resolution. Indeed, this level of resolution is now sufficient to allow the direct detection of frameshifting events in bacterial ribosome profiling data. To see these events, we looked at the best-known example in the literature, the programmed frameshift on the *prfB* gene encoding RF2. When RF2 levels are limiting, ribosomes pause at a stop codon at the 28th codon in the gene and then shift into the +1 frame. Following the frameshift, ribosomes complete the synthesis of the RF2 protein that is encoded in the new reading frame (35). When we split the ribosome occupancy signal into three components, with reads that map to the first, second or third sub-codon position, this frameshifting behavior is evident in models of the *prfB* gene (Figure 6C). Upstream of the frameshift site, the majority of ribosome footprints are cleaved after the second sub-codon position, as expected. But downstream of the frameshifting event, most footprints are cleaved after the third position, consistent with a +1 frameshift. Very few reads map to the first sub-codon position, either before or after the frameshift site. Quantitation of the reading frame upstream and downstream of the programmed frameshift site further supports this conclusion (Figure 6D and E).

### CONCLUSION

Our ribosome profiling analyses of RelE activity *in vivo* reveal dynamic cycles of cleavage, ribosome rescue and initiation that enrich ribosomes at the 5′-end of genes. This model explains the prior observation that RelE cleavage exhibits this unusual polarity. Although it has been known that RelE-cleaved mRNAs are good targets for the tmRNA rescue system (26,36), our data highlight the importance of the tmRNA *and* ArfA systems in the cellular response to and recovery from RelE activity. How cells exit the dormant state following RNA cleavage by type II toxins is not yet clear, but it seems likely that ribosome rescue is essential for this to occur. Antibiotics are beginning to be developed to target the tmRNA rescue pathway (37), raising the possibility of targeting persister cells and, in particular, their reliance on toxins and ribosome rescue systems.

The precision with which RelE cleaves in the A site makes it a valuable addition to the ribosome profiling method, where it can generate ribosome footprints that reveal reading frame for the first time in bacteria. Going forward, our ability to visualize reading frame with the high degree of 3 nt periodicity in these data can be used to search for novel programmed frameshifting sites in bacteria, as has been done with human ribosome profiling (38). RelE may have application outside bacteria as well: it has also been shown to cleave mRNA in ribosomal A sites in eukaryotes and archaea (39). There it should also prove useful in analyses of reading frame and A-site occupancy. Finally, digestion with RelE alone generates mRNA fragments of various length with ends corresponding to ribosomal A sites. If the protocol for cloning mRNA fragments could be adapted to faithfully capture these fragments irrespective of their size differences, it would yield information about position of two ribosomes and the distance between them, not just the position of single ribosomes that comes from the current protocol. Knowing the distance between the A site of two ribosomes may shed light on translational efficiency, rates of initiation, local rates of elongation and ribosome stacking at pause sites.

## ACCESSION NUMBERS

The accession number for the sequencing data reported in this paper is GSE85540.
